# Supplementary material for: PairK: Pairwise k-mer alignment for quantifying protein motif conservation in disordered regions
Source: bioRxiv. 2024 Jul 24:2024.07.23.604860. Preprint. [Version 1] doi: 10.1101/2024.07.23.604860 (PMC11291154; doi:10.1101/2024.07.23.604860)
Supplement: Supplement 1 [file media-1.pdf]

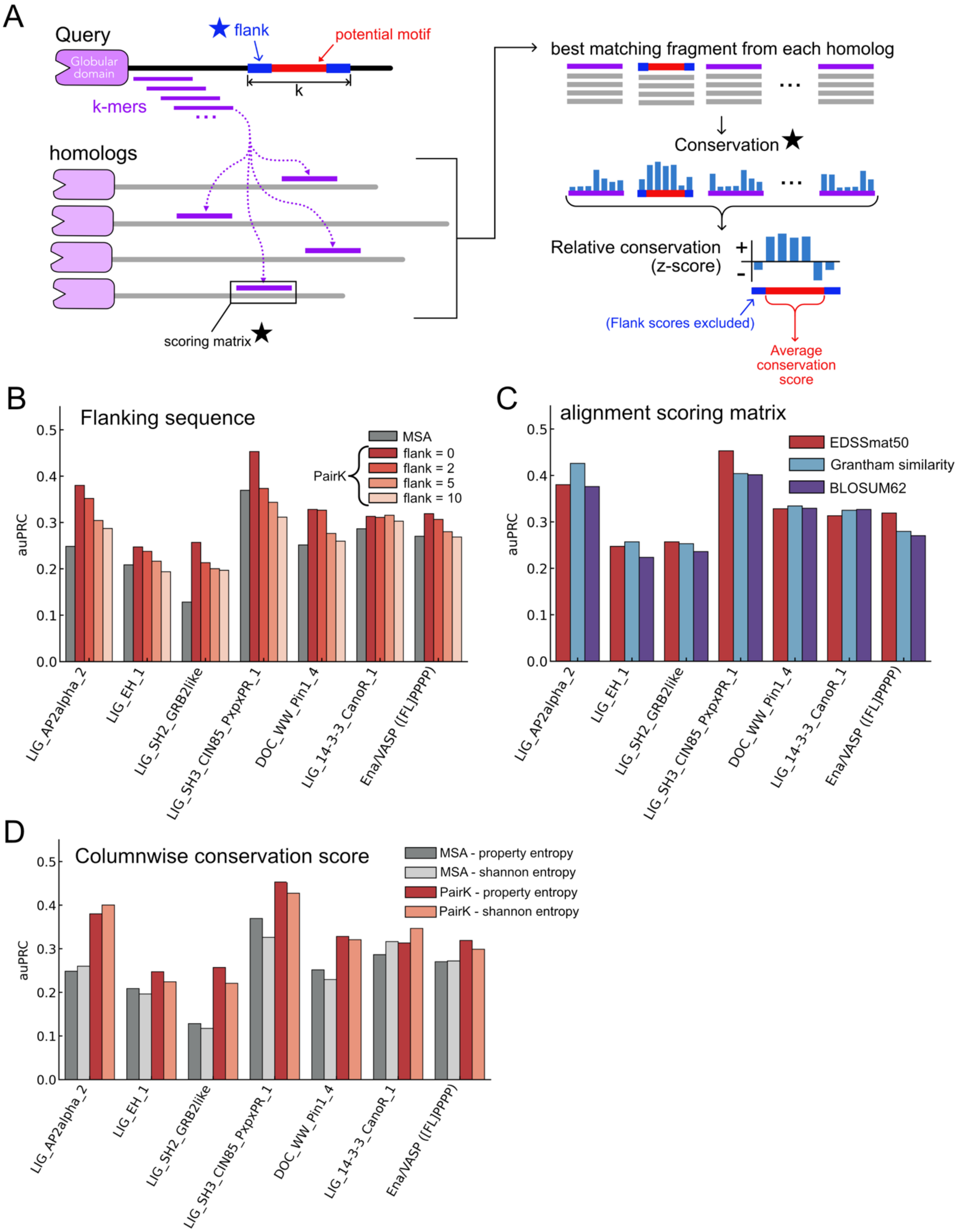

Figure S1. Effect of flanking sequence, scoring matrix, and conservation score method on PairK performance. (A) The PairK method. Stars indicate where the method is changed in parts B-D. The performance of the method is shown when adding residues flanking the potential motif for the alignment step (i.e. increasing k) (B), changing the alignment scoring matrix (C), and using different column-wise conservation scoring methods (D). Column-wise scoring methods used: property entropy and Shannon entropy (*1*). Scoring matrices: Blosum62 (*2*), EDSSmat50 (*3*), and the Grantham matrix converted to a similarity matrix (*4*). Homolog sequences were gathered at the Vertebrata level.

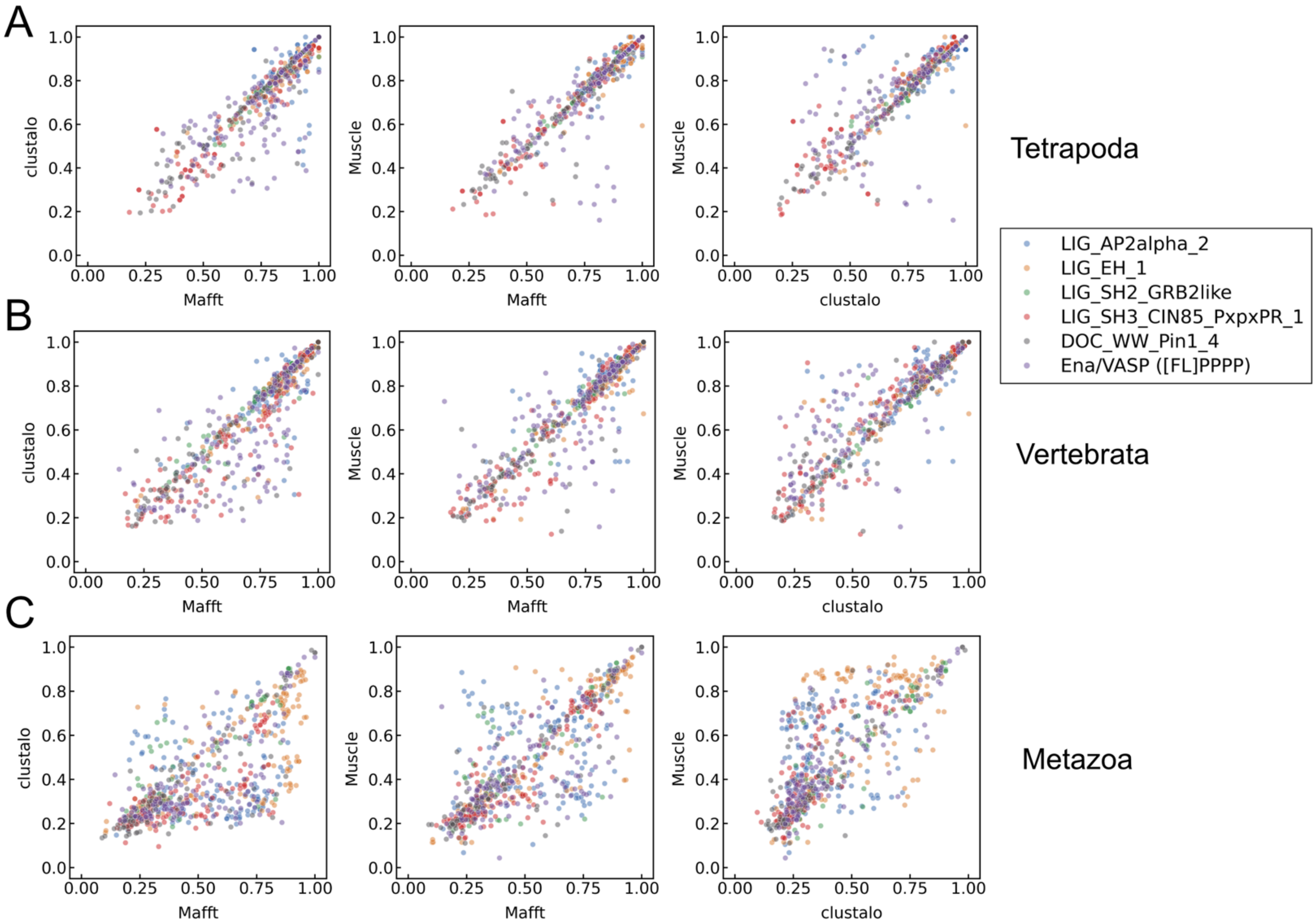

Figure S2. The correlation between conservation scores of residues in experimentally verified SLiMs from MSAs produced by different alignment algorithms. The underlying homolog sequences were collected at the Tetrapod (A), Vertebrate (B), or Metazoa (C) level. Data are from 236 verified SLiM instances (721 residues).

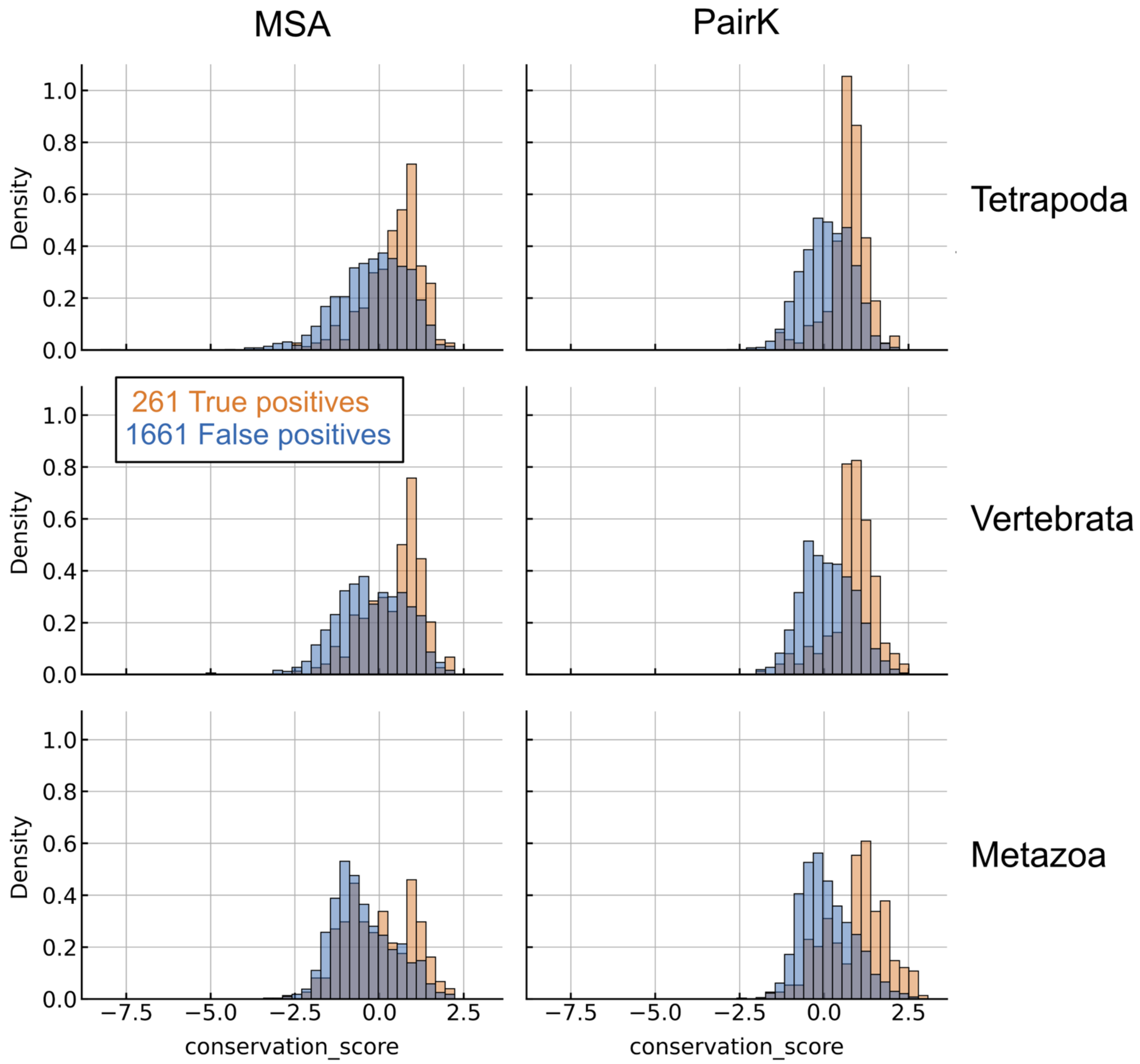

Figure S3. Benchmark conservation score distributions (density) from homolog groups retrieved at the Tetrapoda, Vertebrata and Metazoa levels. True positives are shown in orange and background matches are shown in blue.

Supplementary Tables

Table S1. Counts of SLiM instances in the benchmark for each motif.

SLiM	background	true positives	motif regular expression	mask‡
DOC_WW_Pin1_4	242	42	...([ST])P.	[0 0 0 1 1 0]
Ena/VASP ([FL]PPPP)	241	34	[FL]PPPP	[1 1 0 1 1]
LIG_14-3-3_CanoR_1	246	41	R[^DE]{0,2}[^DEPG]([ST])(([FWYLMV].)|([^PRIKGN]P)|([^PRIKGN].{2,4}[VILMFWYP]))	No mask
LIG_AP2alpha_2	241	49	DP[FW]	[1 1 1]
LIG_EH_1	269	49	.NPF.	[0 1 1 1 0]
LIG_SH2_GRB2like	210	18	(Y)([EDST]|[MLIVAFYHQW])N.	[1 1 1 0]
LIG_SH3_CIN85_PxpxPR_1	212	28	P.[AP].PR	[1 0 1 0 1 1]
TRAF6*	199	7	...P.E..[FYWDE]	[0 0 0 1 0 1 0 0 1]

*TRAF6 was removed from global analyses (*e.g.* auPRC calculations) due to the low number of true positive instances.
‡Positions in the mask array with a 0 were not included in the average conservation score of the motif, whereas positions with a 1 were included. For example, for the sequence DPW and a mask of [1 0 1], the conservation score of the motif would be reported as the average of the first (D) and last (W) residue scores.

Table S2. Manually curated verified Ena/VASP EVH1 binding partners (*5–20*). Interaction data are at the protein level so, for each protein, any FPPPP and LPPPP sequence within an IDR was considered a true positive.

Name	Uniprot ID	reference DOI	OrthoDB id
AB1IP_HUMAN	Q7Z5R6	10.1016/j.devcel.2004.07.021	9606_0:00294e
ABI3_HUMAN	Q9P2A4	10.1016/j.devcel.2014.08.001	9606_0:003dae
ANK3_HUMAN	Q12955	ELM - 10.1093/nar/gkad1058	9606_0:0027f1
FAT1_HUMAN	Q14517	10.1038/sj.emboj.7600380	9606_0:00122c
FBLI1_HUMAN	Q8WUP2	10.1074/jbc.M512107200	9606_0:000661
FYB1_HUMAN	O15117	10.1083/jcb.149.1.181	9606_0:0015fb
LPP_HUMAN	Q93052	10.1091/mbc.11.1.117	9606_0:000d90
NHSL1_HUMAN	Q5SYE7	10.7554/eLife.70680	9606_0:001b40
PALLD_HUMAN	Q8WX93	10.1002/cm.10173	9606_0:00141f
PCARE_HUMAN	A6NGG8	10.7554/eLife.70680; 10.1073/pnas.1903125117; 10.1038/ncomms11491	9606_0:00094d
RAPH1_HUMAN	Q70E73	10.1016/j.devcel.2004.07.024	9606_0:000b76
ROBO1_HUMAN	Q9Y6N7	ELM - 10.1093/nar/gkad1058; 10.1016/s0092-8674(00)80883-1	9606_0:000e6f
SHIP2_HUMAN	O15357	10.1083/jcb.201501003	9606_0:002a4b
SHRM3_HUMAN	Q8TF72	10.7554/eLife.70680; 10.1242/dev.045369	9606_0:0012c3
VINC_HUMAN	P18206	ELM - 10.1093/nar/gkad1058	9606_0:002935
XIRP1_HUMAN	Q702N8	ELM - 10.1093/nar/gkad1058; 10.1016/j.yexcr.2006.03.015	9606_0:000f47
ZYX_HUMAN	Q15942	ELM - 10.1093/nar/gkad1058; 10.1074/jbc.M001698200	9606_0:001d2c

Table S3. Manually curated verified TRAF6 MATH domain interactions (*21–27*).

Name	Uniprot ID	SLiM sequence	reference DOI	OrthoDB id
CD40	P25942	KQEPQEINF	10.1073/pnas.96.4.1234, 10.1074/jbc.274.20.14246	9606_0:004882
TIFA	Q96CG3	SSSPTEMDE	10.1002/cbic.201800436	9606_0:001440
MAVS	Q7Z434	CHGPEENEY	10.1074/jbc.M115.666578	9606_0:00486f
TICAM1	Q8IUC6	CQEPEEMSW	10.1073/pnas.0308496101, 10.4049/jimmunol.171.8.4304	9606_0:004368
IRAK2	O43187	SNTPEETDD	10.1038/nature00888	9606_0:000e31
IRAK1	P51617	PPSPQENSY	10.1038/nature00888	9606_0:004fa3
IRAK1	P51617	PNQPVESDE	10.1038/nature00888	9606_0:004fa3
IRAK1	P51617	RQGPEESDE	10.1038/nature00888	9606_0:004fa3
IRAK3 (IRAK-M)	Q9Y616	PSIPVEDDE	10.1038/nature00888	9606_0:0031e9
mouse TNFRSF11A (RANK)	O35305	RKIPTEDEY	10.1038/nature00888	10090_0:000361
mouse TNFRSF11A (RANK)	O35305	FQEPLEVGE	10.1038/nature00888	10090_0:000361
mouse TNFRSF11A (RANK)	O35305	GNTPGEDHE	10.1038/nature00888	10090_0:000361

Supplementary References

1. J. A. Capra, M. Singh, Predicting functionally important residues from sequence conservation. *Bioinformatics* **23**, 1875–1882 (2007).
2. S. Henikoff, J. G. Henikoff, Amino acid substitution matrices from protein blocks. *Proc Natl Acad Sci U S A* **89**, 10915–10919 (1992).
3. R. Trivedi, H. A. Nagarajaram, Amino acid substitution scoring matrices specific to intrinsically disordered regions in proteins. *Sci Rep* **9**, 16380 (2019).
4. R. Grantham, Amino acid difference formula to help explain protein evolution. *Science* **185**, 862–864 (1974).
5. B. Drees, E. Friederich, J. Fradelizi, D. Louvard, M. C. Beckerle, R. M. Golsteyn, Characterization of the interaction between zyxin and members of the Ena/vasodilator-stimulated phosphoprotein family of proteins. *J Biol Chem* **275**, 22503–22511 (2000).
6. P. F. M. van der Ven, E. Ehler, P. Vakeel, S. Eulitz, J. A. Schenk, H. Milting, B. Micheel, D. O. Fürst, Unusual splicing events result in distinct Xin isoforms that associate differentially with filamin c and Mena/VASP. *Exp Cell Res* **312**, 2154–2167 (2006).
7. T. F. J. Plageman, M.-I. Chung, M. Lou, A. N. Smith, J. D. Hildebrand, J. B. Wallingford, R. A. Lang, Pax6-dependent Shroom3 expression regulates apical constriction during lens placode invagination. *Development* **137**, 405–415 (2010).
8. J. C. Corral-Serrano, I. J. C. Lamers, J. van Reeuwijk, L. Duijkers, A. D. M. Hoogendoorn, A. Yildirim, N. Argyrou, R. A. A. Ruigrok, S. J. F. Letteboer, R. Butcher, M. D. van Essen, S. Sakami, S. E. C. van Beersum, K. Palczewski, M. E. Cheetham, Q. Liu, K. Boldt, U. Wolfrum, M. Ueffing, A. Garanto, R. Roepman, R. W. J. Collin, PCARE and WASF3 regulate ciliary F-actin assembly that is required for the initiation of photoreceptor outer segment disk formation. *Proc Natl Acad Sci U S A* **117**, 9922–9931 (2020).
9. K. Boldt, J. van Reeuwijk, Q. Lu, K. Koutroumpas, T.-M. T. Nguyen, Y. Texier, S. E. C. van Beersum, N. Horn, J. R. Willer, D. A. Mans, G. Dougherty, I. J. C. Lamers, K. L. M. Coene, H. H. Arts, M. J. Betts, T. Beyer, E. Bolat, C. J. Gloeckner, K. Haidari, L. Hetterschijt, D. Iaconis, D. Jenkins, F. Klose, B. Knapp, B. Latour, S. J. F. Letteboer, C. L. Marcelis, D. Mitic, M. Morleo, M. M. Oud, M. Riemersma, S. Rix, P. A. Terhal, G. Toedt, T. J. P. van Dam, E. de Vrieze, Y. Wissinger, K. M. Wu, G. Apic, P. L. Beales, O. E. Blacque, T. J. Gibson, M. A. Huynen, N. Katsanis, H. Kremer, H. Omran, E. van Wijk, U. Wolfrum, F. Kepes, E. E. Davis, B. Franco, R. H. Giles, M. Ueffing, R. B. Russell, R. Roepman, An organelle-specific protein landscape identifies novel diseases and molecular mechanisms. *Nat Commun* **7**, 11491 (2016).
10. M. Krause, J. D. Leslie, M. Stewart, E. M. Lafuente, F. Valderrama, R. Jagannathan, G. A. Strasser, D. A. Rubinson, H. Liu, M. Way, M. B. Yaffe, V. A. Boussiotis, F. B. Gertler, Lamellipodin, an Ena/VASP ligand, is implicated in the regulation of lamellipodial dynamics. *Dev Cell* **7**, 571–583 (2004).
11. M. Boukhelifa, M. M. Parast, J. E. Bear, F. B. Gertler, C. A. Otey, Palladin is a novel binding partner for Ena/VASP family members. *Cell Motil Cytoskeleton* **58**, 17–29 (2004).
12. Y. Zhang, Y. Tu, V. Gkretsi, C. Wu, Migfilin interacts with vasodilator-stimulated phosphoprotein (VASP) and regulates VASP localization to cell-matrix adhesions and migration. *J Biol Chem* **281**, 12397–12407 (2006).
13. M. J. Moeller, A. Soofi, G. S. Braun, X. Li, C. Watzl, W. Kriz, L. B. Holzman, Protocadherin FAT1 binds Ena/VASP proteins and is necessary for actin dynamics and cell polarization. *EMBO J* **23**, 3769–3779 (2004).
14. M. Krause, A. S. Sechi, M. Konradt, D. Monner, F. B. Gertler, J. Wehland, Fyn-binding protein (Fyb)/SLP-76-associated protein (SLAP), Ena/vasodilator-stimulated phosphoprotein (VASP) proteins and the Arp2/3 complex link T cell receptor (TCR) signaling to the actin cytoskeleton. *J Cell Biol* **149**, 181–194 (2000).
15. M. M. Petit, J. Fradelizi, R. M. Golsteyn, T. A. Ayoubi, B. Menichi, D. Louvard, W. J. Van de Ven, E. Friederich, LPP, an actin cytoskeleton protein related to zyxin, harbors a nuclear export signal and transcriptional activation capacity. *Mol Biol Cell* **11**, 117–129 (2000).
16. G. J. Bashaw, T. Kidd, D. Murray, T. Pawson, C. S. Goodman, Repulsive axon guidance: Abelson and Enabled play opposing roles downstream of the roundabout receptor. *Cell* **101**, 703–715 (2000).
17. X. J. Chen, A. J. Squarr, R. Stephan, B. Chen, T. E. Higgins, D. J. Barry, M. C. Martin, M. K. Rosen, S. Bogdan, M. Way, Ena/VASP proteins cooperate with the WAVE complex to regulate the actin cytoskeleton. *Dev Cell* **30**, 569–584 (2014).
18. M. Kumar, S. Michael, J. Alvarado-Valverde, A. Zeke, T. Lazar, J. Glavina, E. Nagy-Kanta, J. M. Donagh, Z. E. Kalman, S. Pascarelli, N. Palopoli, L. Dobson, C. F. Suarez, K. Van Roey, I. Krystkowiak, J. E. Griffin, A. Nagpal, R. Bhardwaj, F. Diella, B. Mészáros, K. Dean, N. E. Davey, R. Pancsa, L. B. Chemes, T. J. Gibson, ELM-the Eukaryotic Linear Motif resource-2024 update. *Nucleic Acids Res* **52**, D442–D455 (2024).
19. E. M. Lafuente, A. A. F. L. van Puijenbroek, M. Krause, C. V. Carman, G. J. Freeman, A. Berezovskaya, E. Constantine, T. A. Springer, F. B. Gertler, V. A. Boussiotis, RIAM, an Ena/VASP and Profilin ligand, interacts with Rap1-GTP and mediates Rap1-induced adhesion. *Dev Cell* **7**, 585–595 (2004).
20. T. Hwang, S. S. Parker, S. M. Hill, R. A. Grant, M. W. Ilunga, V. Sivaraman, G. Mouneimne, A. E. Keating, Native proline-rich motifs exploit sequence context to target actin-remodeling Ena/VASP protein ENAH. *Elife* **11** (2022).

21. H. Ye, J. R. Arron, B. Lamothe, M. Cirilli, T. Kobayashi, N. K. Shevde, D. Segal, O. K. Dzivenu, M. Vologodskaia, M. Yim, K. Du, S. Singh, J. W. Pike, B. G. Darnay, Y. Choi, H. Wu, Distinct molecular mechanism for initiating TRAF6 signalling. *Nature* **418**, 443–447 (2002).
22. S. S. Pullen, T. T. Dang, J. J. Crute, M. R. Kehry, CD40 signaling through tumor necrosis factor receptor-associated factors (TRAFs). Binding site specificity and activation of downstream pathways by distinct TRAFs. *J Biol Chem* **274**, 14246–14254 (1999).
23. N. Tsukamoto, N. Kobayashi, S. Azuma, T. Yamamoto, J. Inoue, Two differently regulated nuclear factor kappaB activation pathways triggered by the cytoplasmic tail of CD40. *Proc Natl Acad Sci U S A* **96**, 1234–1239 (1999).
24. W.-C. Huang, J.-H. Liao, T.-C. Hsiao, T.-Y. W. Wei, M. Maestre-Reyna, Y. Bessho, M.-D. Tsai, Binding and Enhanced Binding between Key Immunity Proteins TRAF6 and TIFA. *Chembiochem* **20**, 140–146 (2019).
25. Z. Shi, Z. Zhang, Z. Zhang, Y. Wang, C. Li, X. Wang, F. He, L. Sun, S. Jiao, W. Shi, Z. Zhou, Structural Insights into mitochondrial antiviral signaling protein (MAVS)-tumor necrosis factor receptor-associated factor 6 (TRAF6) signaling. *J Biol Chem* **290**, 26811–26820 (2015).
26. Z. Jiang, T. W. Mak, G. Sen, X. Li, Toll-like receptor 3-mediated activation of NF-kappaB and IRF3 diverges at Toll-IL-1 receptor domain-containing adapter inducing IFN-beta. *Proc Natl Acad Sci U S A* **101**, 3533–3538 (2004).
27. S. Sato, M. Sugiyama, M. Yamamoto, Y. Watanabe, T. Kawai, K. Takeda, S. Akira, Toll/IL-1 Receptor Domain-Containing Adaptor Inducing IFN-β (TRIF) Associates with TNF Receptor-Associated Factor 6 and TANK-Binding Kinase 1, and Activates Two Distinct Transcription Factors, NF-κB and IFN-Regulatory Factor-3, in the Toll-Like Receptor Signaling. *J. Immunol.* **171**, 4304 (2003).
